# 

**Published:** 1881-05

**Authors:** 


CONTENTS.

| | Page. | | Page. |
|---|---|---|---|
| Neuralgia | 163 | A Physician's Lifetime | 180 |
| Water as a Drink | 165 | Reading | 181 |
| Wealth and Meanness | 166 | House Building | 182 |
| Educated Girls | 167 | Hydrophobia | 183 |
| A Cool Detective | 168 | Climate for Consumptives | 185 |
| Washington's State Dinners | 169 | Carelessness | 186 |
| Climbing the Ladder of Fame | 170 | Wakefulness | 187 |
| Sign of Death | 173 | Health Ruined | 189 |
| Sixteen and Sixty (poetry) | 173 | What Is ? (poetry) | 191 |
| Appetite | 175 | Petroleum in Bronchitis | 194 |
| Decision of Character | 176 | Watering Horses | 19 |
| Be Systematic | 179 | Suppressing the Musquito | 196 |

